# Fever Nursing

**Published:** 1908-02-29

**Authors:** 


### Nursing Administration.

### FEVER NURSING.

### I.—OLD AND NEW STYLE.

Owing to the compulsory notification of infectious disease, fever nursing has gradually risen from being merely a branch of special nursing to a position of immense importance to the community at large. The powers bestowed on municipalities to compel the isolation of persons suffering from infectious fevers have thrown great responsibilities on the heads of the institutions to which the public are consigned willy-nilly, to be nursed at the public expense. Every blunder in treatement or defect in nursing is likely to be bitterly resented by people who have been compulsorily taken from their homes, and it is not surprising that a high degree of skill and administrative ability have been drawn to this field, with a view to overcoming the difficulties which lie in the way of good fever nursing.

The difficulties are exceptional. Save in the cases of some fifteen or twenty large towns in the kingdom, fever hospitals are exposed to so great fluctuations in the number of occupied beds that it is necessary to keep only a small permanent staff, and to engage whoever can be obtained at a moment's notice for times of pressure. For many years highly trained nurses regarded fever nursing with much disfavour, and the attendant secured for ministering to the fever-stricken compared very poorly with the hospital probationer. But during the last few years —perhaps it might more accurately be said the last five years—a definite change has come over the science and art of fever nursing. It has passed from what may be called the "cooling-drink stage," in which the attention of the nurse was mainly concentrated on the effort to make the patient comfortable, to a stage in which the problem is sharply defined, and the duty of the nurse resolves itself into a hand-to-hand conflict with infection. The patients admitted into gigantic institutions receiving every kind of infectious fever, have been found liable to contract from each other fresh forms of infection, and such is the unexpected course of many apparently ordinary complaints that it has proved in practice impossible to keep each specific fever in separate blocks. There must continually be a period of uncertainty during the development of the disease in which the patient may be a source of danger to the inmates of any ward to which he may be assigned.

Out of this condition of affairs has arisen a scientific system of combating infection which has elevated the fever nurse into one of the most skilled members of her profession. The numbers are too great, the cost to the municipality too enormous, to admit of any system of separate wards being adopted. But when once the principle of air-borne infection was abandoned—and we believe that with very few exceptions this principle has now been dropped by all superintendents of fever hospitals in the kingdom—the problem of confining the infectious germs of each patient entirely to his own immediate surroundings became merely a question of exact nursing. The territory on which the bed of each patient rests is to be regarded as the limit of that particular germ's excursions. In order to define this more sharply to the eye, glass cubicles stretching half-way up the ward are in use in some fever hospitals. In others screens separate one kind of fever patient from another. But in the most advanced hospitals it is now found that what is known as the "invisible barrier" is equally effectual in preventing the spread of infection from one person to another. Under this system patients are isolated by means of a simple length of cord. Ordinary acute cases of defined fevers are shut off from the rest of the ward behind a blue cord. Where danger of further developments is suspected, or where the case is of a septic nature, the cord is red, and in such cases the precautions taken are doubled. The unreserved part of the ward is occupied by those who are making a good recovery and show no complications. The nurse's part in the maintenance of the invisible barier is all important. She is constrained to regard each corded-off space as a distinct ward. The great source of infection being the patient's discharges, she is continually and alertly exercised in spraying, in syringing, in swabbing, with a view to preventing the desiccation of any particle of infective matter. She has a special system of bedmaking which enables her constantly to renew any portion of the bedclothes which comes in contact with a dangerous spot. She has a separate equipment within each pen for the patient, so that no article which is touched by one is ever within reach of another. She has the means of personally disinfecting herself within her bounds, and must never issue from the inside of the cord until this has been done. In dangerous cases within the red cords she wears a separate cloak, which she doffs on departure.

This brief outline of the system does not indicate half the delicate and admirably thought-out precautions, on the exact performance of which the success of the fever nurse depends. It will be seen, however, at once, that the methods of the new fever nurse are no whit inferior to those of surgical nurses in the combat incessantly waged against the invading microbe. Sound qualities of brain and long subjection to exact discipline are needed to carry out a system of this complicated nature successfully without chance of a break in the protective barrier. The issue will be interesting to watch. Is this new science of fever-nursing going to revolutionise our treatment of fevers? Will it be possible to isolate, under this system, with the aid of experienced nurses, the sufferers from infectious fevers in their own homes, however crowded the centre? It is premature to speculate on this consummation. But what is certain is that the fever nurse has a great future before her, and may come in time to be able to prove that effectual instrument in eradicating zymotic disease, which the enormously costly establishments of the municipalities have so far conspicuously failed to show themselves.

